# Higher education at the margins – success criteria for blended learning systems for marginalized communities

**DOI:** 10.1007/s10639-022-11282-3

**Published:** 2022-08-25

**Authors:** Anna Mayr, Stefan Oppl

**Affiliations:** 1 Global Headquarter JWL, 18, rue Jacques-Dalphin, 1227 Carouge/GE, Geneva Switzerland; 2 University for Continuing Education Krems, Dr.-Karl-Dorrek-Straße 30, 3500 Krems, Austria

**Keywords:** Blended learning, Higher education programs, Refugees, Success factors

## Abstract

Providing access to higher education for people in marginalized communities, in particular for refugees, requires to re-think the traditional ways of teaching and learning in higher education institutions. The challenges of these circumstances both in terms of access to learning materials and the opportunity to collaboratively learn with others require specific support via appropriate didactical settings. Blended learning arrangements, i.e., settings that bring together online learning activities with synchronous, co-located settings show potential for addressing these requirements. In the present study, we examine the success factors in the design of blended learning settings for supporting higher education in marginalized communities. Based on an established model of blended learning success, we explore the specific challenges of the target group via a survey which was distributed to students of different subject areas and of the higher education programs of Jesuit Worldwide Learning. The 80 survey participants analyzed in this paper live in refugee camps, or marginalized areas located in rural and remote areas in Afghanistan, Guyana, India, Iraq, Kenya, Malawi, Myanmar, Philippines, Sri Lanka and Thailand. While we could confirm the success factors that also apply for blended learning scenarios in traditional settings, we also found evidence for the crucial role of facilitation in both, online and co-located learning phases, and challenges regarding the access to suitable infrastructure. Both need to be considered during design of blended learning programs for this target group.

## Introduction

Access to higher education is particularly challenging for people who are either displaced within their home country or living in exile as refugees (Federal Foreign Office et al., 2019). Globally, 68.5 million people face the challenging living conditions that arise when forced to leave their established living environment (ibid.). According to UNHCRs education report (UNHCR, 2019, p. 13), in particular young refugees are confronted with lacking opportunities for adequate education. There is an evident lack of offers for secondary schooling (ibid.), which leads to a small fraction of this group even meeting the entry requirements for higher education programs. Even if they do, they face even greater challenges. There are hardly any institutions that offer such programs in places that are accessible to refugees (Crea, 2016; Stevenson & Baker, 2018). Restrictions of mobility for stateless persons or national regulations that do not allow refugees to enroll in universities put higher education out of reach for many young refugees. Many of them must work hard to support their families financially, and full-time courses of study, which are usually associated with high tuition fees, are therefore only affordable for a very small fraction. This leads to the fact that only one per cent of refugees enrolled in tertiary education, compared to 37 per cent of young people worldwide (Federal Foreign Office et al., 2019).

Technological evolution and digitalization in recent years offers the potential to mitigate at least some of these challenges. In their strategic report for refugee inclusion, the United Nations refugee agency UNHCR committed to the goal that 15% of refugee youth can access higher education by 2030. To reach this goal, among other measures, UNHCR proposes to strengthen “connected learning” programs as an approach to enable students living in marginalized remote areas to enroll with top universities, by using IT technology and conducting onsite face-to-face learning (UNHCR, 2020b). This approach, which merges online and face-face learning (ibid.), is a specific form of blended learning, where digitally supported learning content delivery is combined with situated direct personal contacts in local peer groups to contextualize the learning process and allow to focus on personal interests, local opportunities and building relationships (Ito et al., 2013). Since 2010, more than 25,000 refugee students spread over 23 countries have been participating in such technology-supported learning programs (UNHCR, 2020a).

In contrast to other blended learning settings, the specific setting faces several challenges that are mainly caused by the living and study conditions the target group faces: First, infrastructure constraints might impact the bandwidth available and connection stability necessary for online learning activities. Second, providers of learning content usually will not be aware of the specific local context and challenges. For connected learning to work, learning processes in the peer group thus need to be facilitated by additional, local staff in a coherent overall pedagogical concept.

Especially during the COVID-19 pandemic, the problem of excluding learners form access to education due to internet connectivity issues was more acute, with interventions such as lockdowns and school closures putting even more learners in remote areas without access to stable internet in situations where alternative pathways for digital learning had to be established. The open-source solution Kolibri was developed by Learning Equality to provide countries like Jordan with a library of learning materials, support and technology during the pandemic to enable digital learning when there is little infrastructure, limited resources of relevant learning materials, and limited support for educators to use technology under challenging circumstances with limited internet connectivity (Akkinepally et al., 2021). The UNESCO Chair in ICT for Development, with support from the EdTech Hub published a report where they mention that there is a need to evaluate carefully how much money was wasted on short-term, out-of-context digital school projects in the wake of the Covid 19 pandemic school closures (Unwin et al., 2020). To avoid such biases when examining the specific requirements of marginalized students on blended learning systems, this article focusses on the offers of Jesuit Worldwide Learning (JWL), an initiative that has more than 10 years of experience with digital higher education approaches in marginalized regions.

The non-governmental organization of the Jesuit Order is an established provider of such learning offers. Crea and Sparnon (2017) reviewed the pilot phase of the project Jesuit Commons: Higher Education at the margins (JC:HEM). The present article takes up the project again and uses its context to examine which aspects of blended learning offers contribute to their success specifically for students in marginalized regions.

JC:HEM has been working since 2010 on the implementation of an online program for higher education for marginalized regions, at the beginning mainly addressing refugees (Balleis et al., 2020). The pilot phase started with programs in Dzaleka camp in Malawi, Kakuma camp in Kenya and urban refugees in the city of Aman in Jordan. By end of 2013, the pilot phase was over, and the capacity building phase of the organization began. To date, the courses continue to evolve, and the organization now operates under the name Jesuit Worldwide Learning. In addition to the liberal studies program that has existed since the pilot phase, a new branch of programs has evolved from the experience of the organization, the Professional Certificate Courses. In addition, the organization expanded its offers geographically to 17 countries. More than 4000 forcibly displaced and other young people from marginalized regions are now enrolled annually.

Based on the experiences of the last years, the pedagogic framework and the course design were further developed into a blended learning concept. After having established the blended learning system based on the findings of Crea and Sparnon (2017) and several years of practical experience, this article revisits this case again to investigate which success factors are specifically important for a blended learning system targeting at higher education students in marginalized regions. We strive to empirically identify the relevant factors by applying an existing instrument to examine blended learning success (Zhang & Dang, 2020) to the specific study context in a field study spanning a diverse group of JWL’s educational offers. While existing research (ibid.) has comprehensively examined the success factors of blended learning in traditional HEI settings, the specifics of studying in marginalized regions as well as the support measures required to enable successful learning under these circumstances have not been examined so far.

The main contribution of this paper addresses thus is the identification of factors relevant for the success of blended learning systems for marginalized communities that are imposed by the specific learning context these communities are confronted with. These findings allow to make informed decisions when designing and further developing learning support infrastructures for this target group.

This paper is structured as follows: First, the topic of learning technologies for higher education at the margins is outlined by explaining the background to the challenges and characteristics of the target group. In addition, an overview of related work in this area is presented to identify the current state of research. To understand the structure and orientation of JWL's blended learning system and thus clarify the context of the field study, the learning system and the foundations of the program are briefly outlined. Subsequently, the methodology of the study is explained, and the results of the study are presented. The discussion links the findings of the current research on the success factors of blended learning systems to the results of our study. The success factors derived from this research, which are relevant from the point of view of the target group, are finally summarized and the further research potential is identified.

## Learning technologies for higher education at the margins

When examining technology-enhanced learning and learning technologies, focus is often put on technical innovations and how they can facilitate learning processes. In the context of educational offers for disadvantaged groups, technology not only can be a facilitator, but can provide the steppingstone to be able to participate in learning at all. However, the living conditions under which participants must pursue their learning aims pose constraints and requirements on the used technology. The focus here often shifts from exploring technical potential and developing new solutions to making existing technology sufficiently stable and available under challenging conditions.

### Challenges of higher education “at the margins”

This paper addresses the factors that determine the success of blended learning systems from the perspective of students in marginalized regions around the world. Only with the challenges of the target group in mind, is it possible to understand the differences in the evaluation of success-critical factors to results which occur in such evaluations in other groups of students.

The term “at the margins” in this paper is used to delineate the research perspective and should in no way represent a discrimination. “At the margins” refers to people, especially youth, at the margins of society, due to poverty, living in low-resource environments and locations, experiencing a lack of opportunities, forced displacement or conflict. Refugees are particularly underrepresented in higher education programs because they face particular challenges in accessing higher education. Many authors address refugees’ challenges for a more equal access to higher education (Brugha & Hollow, 2017; Federal Foreign Office et al., 2019; Gladwell et al., 2016; UNESCO, 2018). Refugees are therefore particularly, but not exclusively, considered in the context of this study.

Looking at the challenges of refugees and people living in marginalized regions in terms of access to higher education as documented in reports, different aspects can be identified: Challenges on the one hand can arise from the education systems in the respective countries, and on the other hand, from the education levels of the students. Examples are the lack of secondary schooling, tuition fees or lack of required English skills (Gladwell et al., 2016, pp. 12–13; UNESCO, 2018, p. 14). These problems are not considered further here, as they have to be addressed at a higher level from international institutions due to their complexity and interconnectedness and cannot be addressed by a learning system. In addition, equal access to higher education programs for youth at the margins is hindered by individual challenges and barriers, such as issues related to distance, special challenges for women, lack of learning communities, and self-confidence (Balleis et al., 2020; Gladwell et al., 2016b, p. 13; UNHCR, 2019, p. 13). If learning technologies should help to overcome some of the challenges and barriers, it is necessary to adapt the learning technologies to the specific requirements. Students in marginalized regions frequently face the lack of reliable electricity or power supply. Furthermore, in many remote rural areas and even in refugee camps, the lack of reliable and continuous internet connectivity challenges students. In addition, a lack of availability and maintenance of appropriate devices for studying should be taken into account (Balleis et al., 2020; UNESCO, 2018, p. 13). The study by Bauer and Gallagher (2020) focuses on the technical challenges that arise when students in refugee camps want to participate in technology-based higher education programs, especially the costs of implementation (which need to be particularly low), the poor connectivity, which is also often associated with costs for data volume, the availability of free power sources and the availability of internet-enabled devices.

In summary, there are numerous barriers for students in marginalized regions to access higher education. When offering higher education programs for students in marginalized regions, it is important to ensure that these programs address the challenges faced by the target group, in particular when technology is used as a support measure (Crea & Sparnon, 2017; Russell & Weaver, 2019). In their study of a higher education preparation program in refugee camps in Uganda, Nanyunja et al. also describe the special consideration of uneven connectivity and capacity when it comes to digital education offerings. In marginalized regions digital education can affect issues such as justice and social inclusion, by excluding students due to inaccessibility of devices or internet connectivity. (Nanyunja et al., 2022, p. 10).

### Educational technology deployment “at the margins”

With respect to scientific exploration of the scientific challenges of technology-enhanced learning for underprivileged people in general and refugees in particular, there are several lines of argumentation.

One line of scientific inquiry explicitly looks at the social justice dimension of educational technology. Numerous authors call for social justice in educational technology sector (e.g., Reinhardt, 2018). The key is to increase research on educational challenges and principles to advance educational technology and create more equitable opportunities for the underprivileged. There is hardly adequate research in the context of e-learning offered in refugee settings (Reinhardt, 2018).

A similar diagnosis is made by Bauer and Gallagher (2020) with respect to the lack of scalable and sustainable solution of higher education programs addressing the educational needs of refugees and the challenging circumstances. While they could identify many efforts and projects in this domain, and the learnings from those efforts are also reflected in the project they reviewed in Southern Uganda, they stress the need for further research, in particular to overcome the logistical barriers of technology-based learning in marginalized situations, such as refugee camps (Bauer & Gallagher, 2020). Further research in this area should also focus on describing in concrete steps and criteria what needs to be done to properly develop sustainable programs for higher education in refugee settings (Abuwandi, 2019, p. 72).

Crea and Sparnon (2017) examined the perspective of online and onsite facilitators on the benefits and challenges of the higher education program for students in marginalized regions. The authors point to the problems of communication between all participants and see regular clear communication between the lead faculty and the onsite and online facilitator as the key to overcoming these problems. In terms of curriculum and learning content, faculty and onsite facilitators interviewed in the research program saw cultural sensitivity in the development of learning materials as critical, especially in addressing gender inequities. But they found it important that onsite facilitators are involved in the learning process as mediators who help to contextualize the content. In the online component of the learning concept, they particularly appreciate the possibilities of technology to give students at the margins the opportunity to interact in a global class and thus oppose social exclusion. The authors emphasize the strengthening of digital skills through the use of technology in learning as well as the positive impact of blended-learning approach with mediated onsite instruction for social inclusion (Crea & Sparnon, 2017).

Brown et al. (2017) examined the facilities, educational and technical needs of higher education in a refugee camp, referring to the case study of a program run by Southern New Hampshire University in the Kiziba refugee camp in Rwanda. The authors see one of the main obstacles to the success of higher education provision in problems with internet connectivity, learning facilities and power supply. In their article, they present several options for effective internet access. These include the possibility of using on-site server capacity to load learning materials during the closing times of the learning centers, in order to save capacity when students are using the internet intensively for their studies by also loading learning materials from the local network. Other factors that emerged from this study that could jeopardise success of higher education in refugee camps include the lack of hardware, the lack of academic skills due to a lack of primary and secondary education, language challenges with English as the language of instruction, security risks for students and staff of the local partners, as well as political barriers. (Brown et al., 2017).

Those learnings are considered in the development of the system architecture of JWL's individual learning technology, the JWL Humanitarian eLearning Platform, JWL-HeLP, which is a crucial part of the blended learning system and will be explained in detail in the following section.

### JWL blended learning model

JWL offers a diverse range of higher education courses for young people aged 18 and above (Jesuit Worldwide Learning, 2020). JWL works with universities and educational institutions worldwide, where universities contribute their subject matter knowledge, professors to accompany students as online facilitators, the accreditation of courses to enable students to reach credits, certificates, and degrees. In the field, JWL works with the Jesuit Provinces and institutions, and other like-minded organizations. The field partners contribute infrastructure and management of community learning centers and accompany students on site by facilitating their learning process. This local setting enables the blended-learning model referred to in this article. Online learning in this setting replaces the input phase of a traditional class with online learning, while collaborative learning phases in the traditional sense take place face-to-face in so-called in-class meetings which take place in the local learning centers. On-site learning phases are the smaller part of the learning process and the online learning phases the larger part (Balleis et al., 2020).

In line with the recommendations derived by Nanyunja et al. (2022), JWLs courses are uniquely developed for the context of students in marginalized regions. Further, JWL follows the recommendation to include staff members of onsite learning centers, like onsite facilitators as well as university partners not only during the run of courses, but also in the program development phase. Therefore, JWL engages them also in early stages of course design and course enrollment processes. With the participation of University Partners as subject matter experts in the course design phase, context-specific blended-learning programs are developed.

These programs follow a pedagogical approach that resembles Kolb’s experiential learning cycle (Kolb, 1984) and uses organizational scaffolds (Van der Pol et al., 2010) to provide the structure necessary to pursue learning activities under challenging circumstances:

The core elements are *Context—Experience—Reflection—Action—Evaluation*. Those elements are reoccurring in each unit of a JWL course. In general, each JWL course consist of 8, 16 or 24 units which are offered as weekly learning chunks. Within a course week the self-study and submission of tasks can be individually adjusted. But each week contains around 20 h of study workload which splits up in self-study learning material and online activities, as well as one onsite meeting with local classmates and an onsite facilitator per week.

#### Context

The life circumstances and the learning situation of the students, thus the "context" of learning, is a central element in the development process of learning content. Case studies and assignments allow continuous reflection on what has been learned and how its related to the individual context. According to the blended learning concept there is not only online studying and submissions but also a weekly onsite meeting that promotes context-related exchange with students in a physical classroom for one hour each week.

#### Experience

During the meeting there are different predefined tasks for teambuilding in the onsite group, but also learning content related tasks in group work or tasks that require an active engagement with the topic in the own community. The strong involvement of an onsite learning group contributes to the contextualization of the content, enables face-to-face interaction, and provides the necessary support and social inclusion. The Global Classroom, where learners can interact and submit their weekly online work, is supervised by a professionally qualified person to ensure quality teaching. Case studies, learning activities and group tasks support a multidimensional, independent acquisition of the learning content. The learning content and assignments are developed specifically for the context of students in marginalized regions in collaboration with subject matter experts from the accrediting university partners and incorporate case studies from the learning communities.

#### Reflection

Regular reflection contributes to a clarification of one's own learning process and a transfer of what has been learned into practice. The on-site facilitators are never subject matter experts and will therefore not deliver subject-related support. Rather, they are trained to support the learning process of the students and are therefore especially concerned to accompany the students and react on individual issues students face while studying and stay in communication with JWL global staff and online facilitators.

#### Action

To ensure the transfer of learning, there must be concrete application situations. The assessed final assignment of each week is designed to take this aspect into account. First, the assignments links content across weeks by applying concepts or artefacts from previous weeks. Each course has a project work component which is thematically different but always aims at a course-parallel application of what has been learned in its own context and is accompanied by reflections and professional support from the online facilitator. The onsite facilitators also play a crucial role in the organizational support for implementation of the project work in the community.

#### Evaluation

Constructive feedback, and comprehensive student support throughout the course enriches the development of the course participants as well as the further development of the course materials and the course itself. Elements of evaluation are found in each section of the course. Self-Check-Quiz chapters are included in the self-study section each units learning materials. Furthermore, feedback and support can be offered on an individual level in face-to-face exchanges during the onsite meetings. In addition, the online facilitator supports student learning process through qualitative feedback for the weekly assessment.

### JWL blended learning infrastructure

**Fig. 1:**
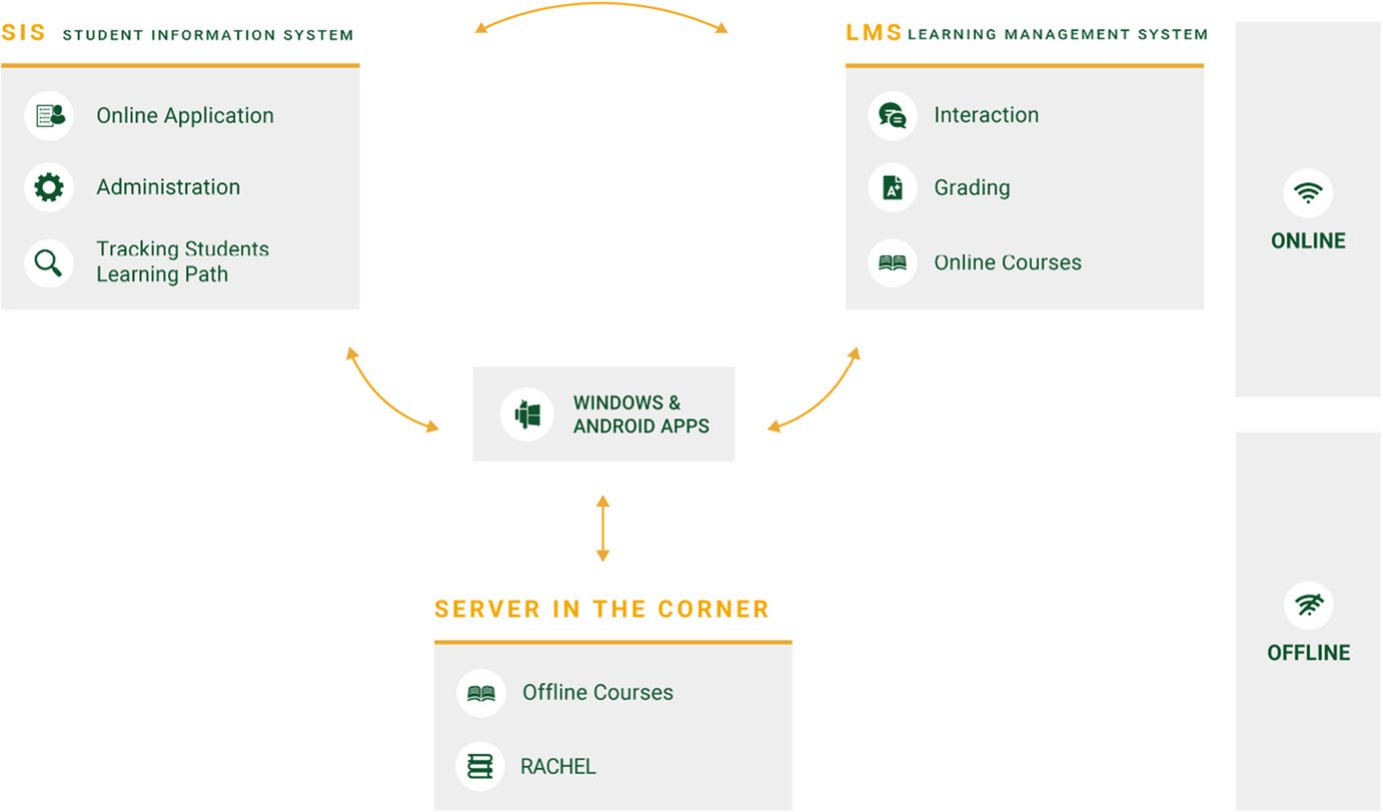
JWL HeLP – Technology to enable the Blended Learning System for students in marginalized regions

In order to implement a blended learning system as described above, a digital learning environment with functionalities such as collaboration of teachers on two levels, the onsite and online facilitator, is required. Onsite facilitators track the attendance of onsite meeting the face-face learning component of the system, while online faculty members evaluate the assessments. Furthermore, the underlying technical system that facilitates studying should also be adapted to the challenges of the learning situation of students at the margins, in particular with respect to instable or low-bandwidth internet connections. One way to counteract this is to have on-site server capacities that load data volumes at times when the load is not high and then enable local distribution of data when required (Brown et al., 2017). Based on this and other specific requirements JWL has started to develop a customized technical system in 2017 that responds to the challenges in the system architecture and is also continuously developed. Figure 1 shows in simplified form how the components *Student Information System*, *Learning Management System*, *Learning Apps* and *Server in the Corner* form the cornerstones of this system. The *server in the corner* is the technical implementation of a decentralized distribution of learning content in a local network to counteract internet connectivity issues. The outsourcing of phases of the learning process that can take place offline, such as the acquisition of learning content and the preparation of activities or projects in the community, to the *learning apps* also reduces internet connectivity issues. The *learning management system* maps the onsite and offline learning situation of the blended learning approach via the role structure with two facilitator roles and a classroom system that is also divided into two parts. The *student information system* enables the enrollment of students from different marginalized regions worldwide in programs of different university partners, through comprehensive management and reporting tools that have been developed specifically for this purpose.

**Fig. 2:**
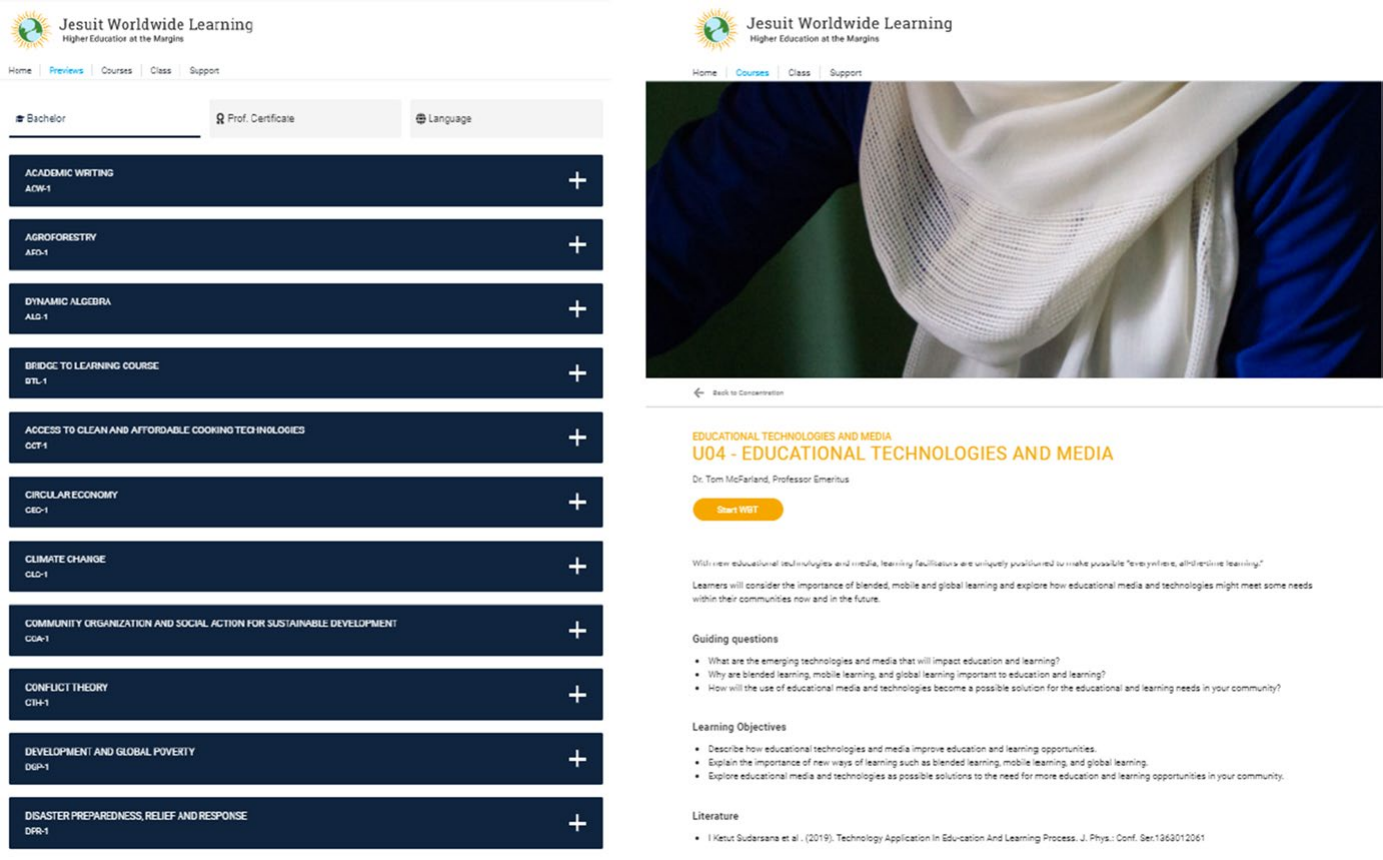
JWL HeLP – sample course list (left) and sample learning content (right)

The term *blended-learning system* in the context of this article always refers to the entire system and includes the technical system with all the components visualized in Fig. 1 as well as organizational system parts like support and instructional design elements (as shown in Fig. 2 for illustrative purposes). This also means the structuring of courses in 8 weeks, as well as the division of each learning week into input acquisition phase, graded online learning activities, reflection tasks, onsite group work in the meetings in class, online discussion, and weekly assignments. The pedagogical foundation of this approach as well as the experience and research of the Diploma courses and their evaluations described in detail by Crea and Sparnon (2017).

From a technical perspective, the offline components of the learning support system are implemented via an appliance referred to as SAINT (Standalone Accesspoint for Infrastructure, Networking and Transmission). SAINT is a laptop which is used as a “server in the corner” and the offline learning apps are the system components that make it possible for the students to access the learning material hosted there.

Consequently, connection to the SAINT does not require internet connectivity. Once a SAINT is turned on in a Community Learning Center and students are in the nearby area, they can connect via WiFi and access all learning material provided by the SAINT through the JWL Learning apps. This helps to overcome the challenge of low connectivity. The IT solution is developed to outsource as many phases of the digital learning process in an offline setting as possible and only connects to the internet when necessary.

The SAINT attempts to download all course data and thus packages of learning content from the global JWL servers every two hours. The apps automatically detect if they are connected to a WiFi with a SAINT present and will then download the course data from there, avoiding the need of individual online connections that would put huge strains on the usually limited internet bandwidth.

In addition, mechanisms are available to enable learning for students who do not have unconstrained access to Community Learning Center, which is an issue in particular for women in some of the target countries. To enable them to continue their studies, it is crucial to be able to access the learning content in small, decentralised learning groups, and without having to walk long distances to and from the learning centre. In such situations, students can be provided with an SD card which contains encrypted packages of learning content (only accessible via the JWL app). Course content is accessible by inserting the SD card in an Android device with the JWL Global e-Learning App installed (with offline studying setting enabled). Any student or facilitator who has downloaded course material at the learning center can export it to a USB stick or SD card and share it with other JWL students in remote rural areas. This allows for the distribution of learning content to students’ mobile devices without the need for an internet connection and enables them to pursue their studies, even in the most challenging contexts.

## Method

The aim of this article is to identify the specific requirements of marginalized students on blended learning models and their technical support systems. We therefore examine established research models that allow to assess success criteria for blended learning systems and apply them to the specific target group.

### Literature review on blended learning success models

We conducted a literature review to identify a validated model on blended learning success factors building on recent conceptual developments, fitting the context of the case study and at the same time considering the findings of previous studies. From a conceptual perspective, blended learning approaches are social settings in which e-Learning systems are deployed in combination with presence-based learning activities, as is also the case in the JWL scenario described above. We thus here take an inclusive approach to identify relevant factors for blended-learning success and first present related research that adopt a technically oriented perspective on blended learning success (mainly focusing on the e-learning component), before we discuss pedagogically grounded approaches. We finally present studies that have attempted to adopt an integrative perspective and use our findings from the initial perspectives to select an evaluation model that adopts an inclusive perspective on blended learning success.

#### A technical perspective on blended-learning success

E-Learning systems are often considered as specialized instances of information systems (Al-Fraihat, 2019) and are consequently evaluated using models developed and validated in this context (e.g., DeLone & McLean, 2003). The widely deployed IS success model proposed by DeLone and McLean (2003) provides a sound starting point for a blended learning success model, as it has already been used and validated in the context of e-learning success multiple times (Al-Fraihat, 2019).

Petter et al. (2013) identify the independent variables impacting IS success. Task, user, social, project and organizational characteristics should be measured in detail (ibid.), but are not as elaborated in the D&M IS Success model as in other studies. However, the user perspective influenced by external social, cultural or political factors is an important item in the technology acceptance model (TAM) (Davis et al., 1989) and the models building upon it. Many researchers follow the call to focus also on measuring the independent variables. Hence combinations of TAM and the D&M IS success model can be found in many studies on the success of e-learning systems (Al-Fraihat, 2019) to also address human factors and social change related factors while evaluating the acceptance of an e-learning system (ibid.). In the context of technology acceptance of e-learning systems, several studies have identified factors that are relevant for assessing e-Learning success: Media richness (Liu et al., 2005), course design or design of learning material (Ain et al., 2016; Almaiah & Alyoussef, 2019; Lee et al., 2009; Mahande & Malago, 2019), computer anxiety (Abdullah & Ward, 2016; Chen & Tseng, 2012), computer self-efficacy (Abdullah & Ward, 2016; Chen & Tseng, 2012) and in analogy to the findings of the D&M IS success model the facilitator characteristics (Ain et al., 2016; Almaiah & Alyoussef, 2019; Mahande & Malago, 2019).

#### A pedagogical perspective on blended learning success

From a more pedagogically motivated perspective, the HELAM model was one of the first models to bring together technical aspects and social aspects of digital learning in one research framework (Ozkan & Koseler, 2009). The Blended e-Learning Success model (Wu et al., 2010) focusses even more on social aspects of the learning environment. It explicitly considers the impact of didactical aspects in the blended learning context and in particular shows that the learning environment which includes technical aspects as well as social environmental aspects influences the learner’s behavior and learning climate. (Wu et al., 2010).

The impact of didactical principles in the blended-learning design has also been considered in the framework proposed by Lin and Wang (2012). The connection between didactics and IT support was operationalized via the Task-Technology Fit construct. In the evaluation of the framework, this construct showed significant impact on overall blended learning success (Lin & Wang, 2012).

Other non-technical influencing factors that could be identified to be relevant in a study conducted by Al-Busaidi (2012), are classmate characteristics and course flexibility as well as organizational factors. Among the latter, the influence of intensive training for the teachers was particularly positive (Al-Busaidi, 2012). Ghazal et al. (2018b) also underlines the significance of classmate interaction and facilitator training as factors influencing blended learning success.

#### An integrative perspective on blended learning success

More recently, several studies have adopted a holistic approach to blended learning success criteria, considering several of the influence factors identified above in an integrated framework. We could identify four recently proposed frameworks that were analyzed for appropriateness to be deployed in the context of higher education at the margins.

Ghazal et al. (2018a, b) evaluated critical factors to learning management system acceptance and satisfaction in a blended-learning environment. The framework includes aspects of technology acceptance (Davis et al., 1989) as well as information system quality dimensions (DeLone & McLean, 2003). Social and human factors as well as the pedagogical aspects of the course design, emphasized by the other authors, however, were hardly taken into account in this evaluation model (Ghazal et al., 2018a, b). It thus was not considered further for use in the present study.

The model proposed by Alomari et al. (2020) to investigate the human factors influencing the effectiveness of an LMS includes a very broad spectrum of different perspectives of influence, and in particular the focus on human factors. The framework, however, explicitly focusses on the study of effectiveness, which leaves out aspects of acceptance, which were found to be relevant for success of blended-learning systems in earlier studies.

The research model of Seman et al. (2019) mainly focusses on the impact of the deployed learning management system in the blended-learning program but hardly accounts for organizational aspects of the blended learning setting, which are specifically relevant in the deployment setting “at the margins” focused on in this study.

**Fig. 3:**
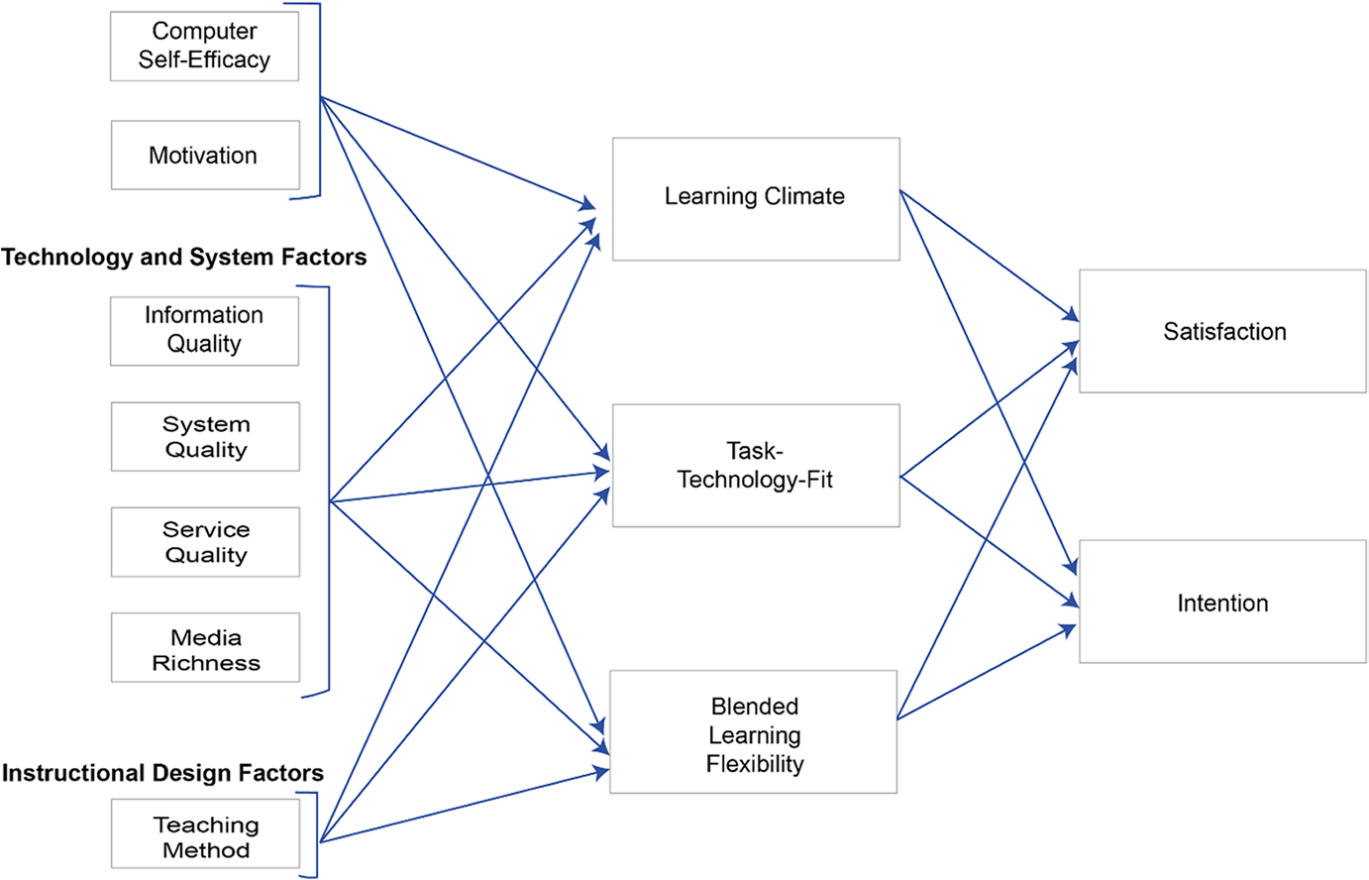
Blended learning success model (adapted from Zhang & Dang, 2020)

Zhang and Dang (2020) have proposed a comprehensive blended learning success model that considers all perspectives identified as potentially relevant above (self-related factors, technology, and system factors, as well as instructional design factors). Furthermore, the model was designed and tested in a similar setting as implemented in the case study reviewed here, evaluating a blended-learning program which includes weekly face-to-face meetings of learner groups as well as online self-study phases with interactive, multimedia learning materials. Summarizing, most of the identified models focus on either the technical aspects or the pedagogical aspects of blended learning success. To take an inclusive approach, we have opted to adopt to model with the broadest scope of examination, which is the Blended-Learning Success Model as proposed by Zhang and Dang (2020), which we present in more detail in the following. Their research model considers blended learning success factors at three levels. As shown in Figs. 3, the authors have grouped the external variables on a first level: the target group-focused perspective with self-related factors, the technology and system-related factors, and the instructional design factors. At the intermediate level of the model, the influence of concepts measured in the e-learning context is modeled. It represents the interplay of aspects of the learning process in the blended-learning environment, including the learning climate, the task-technology fit and the blended-learning flexibility. On the third level, the factors that influence the success of a blended-learning system in the sense of the research question can be found: satisfaction with the system and the intention of the students to use the blended-learning system for further courses are indicators for the blended-learning system's success (Zhang & Dang, 2020).

### Data collection method

To evaluate the blended learning success criteria in the case-study context, the questionnaire proposed by Zhang and Dang (2020) was used for the quantitative part of the study. While it has been deployed and validated in a traditional HEI context, its theoretical foundations are in line with those of the study presented here. The different contexts of application of the instruments (traditional HEI vs. HEI “at the margins”) furthermore allow to identify differences in the results, potentially pointing at aspects that are particularly relevant in the contexts examined here. Minor adaptations to the questionnaire were made to match the nomenclature used in the JWL context without altering the intent of the respective items (cf. Annex 1). According to Zhang and Dang (2020), the items to evaluate the teaching method are highly contextualized within each case study. Therefore, specific items were developed to assess the different aspects of the teaching method in the JWL context. In total there were 67 questions split into 15 topics, where all items were rated on a 5-point Likert scale (*strongly agree* to *strongly disagree*) with an option to select “*prefer not to say*”.

To ensure to capture all aspects crucial from the perspective of students in marginalized regions, participants were additionally given the opportunity to provide freely formulated feedback and outline potential for improvement, which was examined qualitatively. All answers were examined and categorized according to the success factors that were addressed in the research model.

The research model selected for this purpose, as well as most research models included in the literature analysis which led to this selection, were based on evaluations in developed countries. Since the research is conducted using the already existing research instrument of Zhang and Dang (2020), it could be possible that factors are not considered which are crucial from the perspective of students in marginalized regions, but which are not evaluated via the instrument that was designed in developed countries. To ensure to capture all aspects crucial from the perspective of students in marginalized regions, participants were additionally given the opportunity to provide freely formulated feedback and outline potential for improvement, which was examined qualitatively to identify influencing factors they mention without being prompted. For this reason, an open question was included at the end of the questionnaire to find out what improvement potential the students see. The sub-question was: "Do you have any further ideas how JWL Courses and Learning Platform can be improved?” The open question format avoids directing the students to a specific category of success factors and thus prompting certain responses.

Before starting the survey, a pre-test was conducted with six students from three different countries of origin (Afghanistan, Malawi and Northern Iraq) was conducted to assess if the understandability of the study for the target group, which only led to minor adaptations in the terminology used to refer to the blended learning system.

### Data analysis method

The research model underlying the quantitative part of the study already contains a set of variables that were shown to be relevant to describe blended learning success and their potential relationships. In our analysis, we have assessed the results of the survey with respect to both aspects. Descriptive statistics were used to characterize the survey results for each of the variables contained in the research model. Pairwise significance tests were used to check for potentially relevant differences in the results for the variables that would require further examination. In addition, the demographic parameters of gender and age were used to segment the sample and check for different perceptions in the resulting subgroups. In replicating the assessment of relationships among variables conducted by Zhang and Dang (2020), we calculated a correlation matrix and the structural equation model (using SmartPLS 3) to examine the variables’ interdependencies and to enable checking for differences to the results obtained when examining a traditional HEI setting, as has been done in the original study by Zhang and Dang (2020).

For the qualitative part of the study, the answers provided by the students to the open question were coded using the thematic analysis approach proposed by Braun and Clarke (2006). This approach allows to systematically identify themes in a corpus of text using both, deductive and inductive coding steps. To assess whether the qualitative data supports the results of the quantitative part of the study, the answers were thematically coded using a deductive approach following the categories used in the research model of Zhang and Dang (2020). As argued above, constraining analysis to these categories might lead to ignoring potentially relevant additional aspects of blended learning success that are important for the specific target group. Thus, in a second round of analysis, the answers were coded inductively to derive additional themes that might be candidates for further relevant success factors.

### Implementation of data collection

An online survey was sent out to students of JWL´s professional certificate programs in several locations worldwide to evaluate the success criteria of blended-learning systems from a global perspective. This cross-sectional study approach was chosen to account for different challenges that might arise for students in different countries.

The examined courses had been chosen as they show a consistent framework of blended learning, with online individual self-study time, two in-class meetings per week and several online learning activities and assignments. Overall, 450 students had completed these courses or were in the process of doing so, and thus in principle would have been eligible to participate in the study. At the time of the evaluation, 273 of these 450 students from the countries Afghanistan, Guyana, India, Iraq, Kenya, Malawi, Myanmar, Philippines, Sri Lanka and Thailand were still active on the learning platform, the other students already had completed their courses. To reach a group of participants as diverse as possible, an opportunistic sampling approach had been chosen. In accordance with the JWL Research Team, which checked and approved the study from an ethical perspective, the survey was sent out to all coordinators of the involved local learning centers, who distributed the survey link to all students who had participated or still participated in one of these courses. Further notifications were sent out within the learning platform to reach all students who were still active on the learning platform. Participation was voluntary and did not grant any benefits for students. The questionnaire was open for participation between the beginning and the end of October 2020. During this time, 81 complete questionnaires were submitted. One questionnaire was invalid, as the participant did not belong to the target group. Thus, in total, 80 surveys were included in the evaluation. The questions on service quality (SQV) were prefaced by a filter question in which students could indicate whether they had ever used the service, in this case the helpdesk. Only those students who had used the helpdesk were asked further questions about this factor, thus resulting in a total of 18 students.

The demographics of the participants is visualized in Table 1. Half of the questionnaires were completed by women, so the results can be considered as gender balanced. The median age of participants was 22–24 years old. Almost half of the respondents were refugees, whereas the remainder is distributed over other target groups from the host communities of refugee camps. Almost 70% of the students who participated in the survey had taken their first program with JWL, the remainder were returning students, who already knew the blended learning format but had not previously used the newly deployed blended learning system.

**Table 1:** Demographics of participants

	Frequency	Percentage
Gender		
Male	40	50,0
Female	40	50,0
Age		
18–21	16	20,0
22–24	25	31,3
25–29	19	23,8
30–34	11	13,8
35–39	7	8,8
40 and older	2	2,5
Status		
Refugee	39	48,8
Host Community	15	18,8
Student of Jesuit School / Loyola Campus	15	18,8
Other	4	5,0
Prefer not to say	7	8,8
Pre-Experience with JWL		
1st Enrollment	55	68,8
had already been enrolled	24	30,0
Other	1	1,3

The study was affected by the global Corona pandemic, in particular by restrictions regarding face-to-face presence components. 48% of students actively enrolled in a program indicated that the in-class meeting was predominantly face-to-face. 46% indicated that the meeting took place primarily via video conference. For the remaining 6%, the mode changed from presence to video conference. Comparing these results with previously acquired data of students who participated in the programs before 2020, it can be seen that before the pandemic 79% of the in-class meetings took place in presence in the learning centers and only 21% of the students participated in the exchange with their local learning group via video conference.

## Results

In the following, we summarize the results of the quantitative and the qualitative components of our study before discussing and contextualizing the findings in Section 5.

### Quantitative results

The overall results of the questionnaire study are given in Table 2 (scale ranging from 1 to 5, where 5 represents positive perceptions). In summary, all factors are rated positively in general and show no statistically significant differences in pair-wise comparisons as well as for subgroups constituted by gender or age. The factors *intention* and *satisfaction* situated on the outcome-oriented third level of the research model received the highest approval from the students in the survey. The factors *motivation* and *teaching method* also received above-average approval ratings. The factors *learning climate*, *service quality*, *information quality* and *task-technology fit* were rated slightly lower. The statement on the ease of understanding the information was rated worst (IQ2). Due to the low number of respondents for *service quality*, the validity of the values is limited. The *system quality* also scored slightly lower than the other factors on level 1 of the research model. This can be attributed to the relatively low rating of the response time of the system. In the variances, especially the *media richness* and the system quality showed a low spread of the answers, while the *computer self-efficacy*, *service quality*, *blended-learning flexibility* received very broadly spread answers from the students. The importance of in-class meetings is where student opinions diverge the most, although the item (TM6) was still rated highly with a mean of 4.26.

**Table 2:** Descriptive Statistics

Construct	N	Mean	StdDev	Item	Mean	StdDev
CSE Computer Self-Efficacy	80	4,2250	0,6951	CSE1	4,2025	0,8679
				CSE2	4,2625	0,8964
				CSE3	4,2278	0,7671
M Motivation	80	4,2979	0,5516	M1	4,3766	0,5390
				M2	4,2727	0,7370
				M3	4,2911	0,7188
IQ Information Quality	80	4,1027	0,4914	IQ1	4,1923	0,7218
				IQ2	4,0000	0,7463
				IQ3	4,1899	0,8088
				IQ4	4,0897	0,7383
				IQ5	4,0395	0,5233
				IQ6	4,1688	0,7857
SQ System Quality	80	4,2013	0,4521	SQ1	4,1375	0,7070
				SQ2	4,4375	0,5703
				SQ3	4,1039	0,6994
				SQ4	4,3375	0,6353
				SQ5	4,0253	0,7333
SQV Service Quality	18	4,1444	0,6679	SVQ1	4,0556	0,8726
				SVQ2	4,1765	0,6359
				SVQ3	4,1667	0,7859
				SVQ4	4,1176	0,7812
				SVQ5	4,2222	0,6468
MR Media Richness	80	4,2323	0,4869	MR1	4,1250	0,7693
				MR2	4,3125	0,4928
				MR3	4,2250	0,7287
				MR4	4,2692	0,5738
TM Teaching Method	80	4,2753	0,4912	TM1	4,1519	0,6620
				TM2	4,3038	0,5851
				TM3	4,2125	0,6501
				TM4	4,3375	0,7622
				TM5	4,2911	0,7537
				TM6	4,2625	0,8964
				TM7	4,3625	0,6412
LC Learning Climate	80	4,1813	0,5277	LC1	4,1750	0,5905
				LC2	4,2000	0,7187
				LC3	4,2375	0,6212
				LC4	4,1392	0,6351
TTF Task-Technology-Fit	80	4,0917	0,6296	TTF1	4,1625	0,7368
				TTF2	4,0779	0,8073
				TTF3	4,0641	0,8730
				TTF4	4,1818	0,5787
BLF Blended Learning Flexibility	80	4,2188	0,6515	BLF1	4,2692	0,7328
				BLF2	4,1667	0,8283
				BLF3	4,2405	0,7019
SAT Satisfaction	80	4,3552	0,5707	SAT1	4,4500	0,5489
				SAT2	4,3924	0,6287
				SAT3	4,3846	0,6493
				SAT4	4,2152	0,8574
INT Intention	79	4,5000	0,5547	INT1	4,5256	0,5749
				INT2	4,4684	0,5957

As this article strives to identify impact factors on blended learning success in the context of higher education at the margins, we also review the data for potentially correlating factors. Table 3 provides an overview of the correlation results. Since none of the variables is normally distributed, the Spearman correlation coefficient was used for the data analysis. Except for the factors *Computer Self-efficacy* and S*ervice Quality*, there are only significantly positive correlations with other success factors. For these two factors, mostly weak, non-significant correlations could be identified. Only the factors *Task-Technology Fit*, *Information Quality* and *System Quality* show significant correlations with both of those factors.

**Table 3:** Spearman´s Rho correlation coefficients in research model

Spearman Correlation	INT	SAT	BLF	TTF	LC	TM	MR	SVQ	SQ	IQ	M	CSE
INT	1											
SAT	0,516**	1										
	0,000											
BLF	0,539**	0,561**	1									
	0,000	0,000										
TTF	0,323**	0,641**	0,552**	1								
	0,004	0,000	0,000									
LC	0,402**	0,568**	0,493**	0,606**	1							
	0,000	0,000	0,000	0,000								
TM	0,480**	0,656**	0,638**	0,609**	0,587**	1						
	0,000	0,000	0,000	0,000	0,000							
MR	0,431**	0,426**	0,338**	0,524**	0,496**	0,566**	1					
	0,000	0,000	0.002	0,000	0,000	0,000						
SVQ	0,371	0,474*	0,380	0,708**	0,229	0,497*	0,936**	1				
	0,130	0.047	0,120	0.001	0,360	0.036	0,000					
SQ	0,358**	0,436**	0,475**	0,603**	0,501**	0,530	0,565**	0,734**	1			
	0.001	0.000	0,000	0,000	0,000	0,000	0,000	0.001				
IQ	0,274*	0,489**	0,384**	0,573**	0,549**	0,545**	0,519**	0,524*	0,599**	1		
	0,014	0,000	0,000	0,000	0,000	0,000	0,000	0.026*	0,000			
M	0,411**	0,501**	0,525**	0,427**	0,389**	0,559**	0,391**	0,423	0,330**	0,467**	1	
	0.000	0,000	0,000	0,000	0,000	0,000	0,000	0,080	0,003	0,000		
CSE	0,183	0,140	0,213	0,259*	0,320**	0,178	0,266*	0,508*	0,376**	0,333**	0,190	1
	0,107	0,216	0,058	0,020	0.004	0,115	0,017	0,031	0,001	0,003	0,092	

**. correlation is significant at the 0.01 level (two-tailed)

*. correlation is significant at the 0.05 level (two-tailed)

In addition to the correlation matrix, we also calculated the structural equation model testing the hypotheses in relationships among the variables replicating the procedure described in Zhang and Dang (2020) using the PLS method. The results are given in Table 4. The R-squared values are reported in Table 5, indicating to which amount the significant independent variables explain the variance of the respective dependent variables.

**Table 4:** Path hypothesis test results

Path	Path Coefficient	T-Statistics	p-Values
CSE—> LC	0,141	1,583	0,114
M—> LC	0,049	0,287	0,774
CSE—> TTF	0,022	0,295	0,768
M- > TTF	-0,009	0,080	0,936
CSE—> BLF	0,076	0,850	0,396
M—> BLF	0,168	1,318	0,188
IQ—> LC	0,306	1,860	0,063
SQ—> LC	-0,007	0,037	0,971
SVQ—> LC	-0,120	1,052	0,293
MR—> LC	0,109	0,734	0,463
IQ—> TTF	0,262	2,254	0,025*
SQ—> TTF	0,260	1,879	0,061
SVQ—> TTF	0,091	1,126	0,261
MR—> TTF	0,024	0,194	0,846
IQ—> BLF	-0,125	0,978	0,329
SQ—> BLF	0,209	1,658	0,098
SVQ—> BLF	0,029	0,329	0,743
MR—> BLF	-0,164	1,041	0,299
TM—> LC	0,356	2,915	0,004*
TM—> TTF	0,327	2,783	0,006*
TM—> BLF	0,545	3,379	0,001*
LC—> SAT	0,428	3,791	0,000*
TTF—> SAT	0,310	2,690	0,007*
BLF—> SAT	0,086	0,644	0,520
LC—> INT	0,269	1,971	0,049*
TTF—> INT	-0,053	0,329	0,742
BLF—> INT	0,364	2,915	0,004*

*. significant at the 0.05 level

**Table 5.** R-squared values

Construct	R-Squared
LC	0,496
TTF	0,592
BLF	0,418
SAT	0,508
INT	0,250

### Qualitative results

Of the 80 students who filled in the survey, 61 wrote an additional comment. In these comments, satisfaction with the system was explicitly expressed by 35 participants. Their intention to take further courses in the JWL blended learning system was expressed by 29 participants. When deductively coding the free responses and suggestions for improvement (cf. Annex 2 for the comprehensive list of deductively coded responses) to see if success factors from Zhang and Dang (2020)'s model were addressed, it was found that none of the Self-Related Factors were addressed, but all of the Technology and System Factors as well as the Instructional Design Factor, Teaching Method were addressed. Looking at this result in relation to the research model, it is noticeable that the external influence factors are addressed by the students, as well as the endpoints Satisfaction and Intention. However, the dependent constructs Learning climate, Task-Technology-Fit and Blended-Learning Flexibility were not addressed in the free responses.

In addition to the success factors from Zhang and Dang's model, inductive coding (cf. Annex 2 for the comprehensive list of inductively coded responses, representative samples included below for illustrative purposes, references to IDs linking to Annex 2 given in square brackets) allowed to identify that students also addressed the facilitator characteristics which are considered as significant blended-learning system success factors in several previous studies. As within JWL there are two quite different facilitator roles the results are summarized for online facilitator characteristics and onsite facilitator characteristics. Related to the online facilitator the results indicate that from the perspective of students in marginalized regions, the aspects *online facilitator support* (“My online facilitator Ms Tsoi should be appreciated and regarded, for the support she rendered.” [ID64]), *online facilitator qualification* (“I would strongly suggest […] to check the knowledge of all the online facilitators before giving them a class to handle, as choosing weak online facilitator […] can bring the quality of the lessons very low […]” [ID50]), *online facilitator response timelines* (“[…] once we get stuck or have questions, we write questions to the online facilitators but no replies. […] weekly works which are to be marked by the online facilitators are delaying with marking them. […]” [ID51]), and *online facilitator feedback quality* (“There should be an active collaboration between students and online facilitators because they don't give feedback on weekly submitted works. students cannot be able to know if they are doing the works correctly” [ID139]) impact the success of the blended-learning system.

Seven statements clearly show the relevance of the onsite facilitator related aspects which also have an impact on the perceived satisfaction with the system and thus on the success of the blended-learning system. The points mentioned could be grouped into the following arguments. The *onsite facilitator support* (“[…] specially I am thankful from Ms. Shallyn. She is a very kind and understandable girl” [ID25]) and the *qualification of the onsite facilitator* (“[…] our onsi[te] facilitator is very weak and doesn't have enough knowledge about the subject” [ID47]) have a crucial impact, as does the *moderation of the in-class meeting* (“the InClass meetings don't fulfil my learning expectations because everything discussed are quite basic in nature […]” [ID]) and the *helpfulness*, *guidance*, and *training to use the system* (“[…] the course facilitator didn't guide properly and for the first two weeks we were not aware of how to access the course contents […]” [ID53]).

Furthermore, from the results of the free responses it could be recognized that from the perspective of the students in marginalized regions, the equipment with hardware and internet connectivity is also seen as a success factor of a blended-learning system. The topic of hardware and internet connectivity was mentioned six times. Firstly, the students addressed the accessibility and provision of hardware (“You may g[i]ve us big lap top which are new” [ID37]), from which the aspect of accessibility of hardware for studying can be derived. Other students saw a possibility for improvement of the Blended-learning systems with the availability of suitable hardware (“If we could have […] any tablet to watch the videos offline it will be great” [ID140]). Therefore, the aspect fit of available hardware for the requirements in the learning system can be derived. But also, the influence of the internet connectivity, which was mentioned (“[…] it would be better if your center provide[s] a better internet connection.” [ID115]), should not be neglected, resulting in the aspect accessibility of internet connectivity that fulfills the requirements of studying with the blended-learning system.

## Discussion

The importance of a blended-learning approach is supported by a large body of recent literature on the success criteria of e-learning systems for deployment with marginalized target groups. The design of blended-learning approaches and their fit to the context of deployment have significant impact on student satisfaction (Alomari et al., 2020; Seman et al., 2019; Zhang & Dang, 2020).

The results of the present study also support this argument. As both, satisfaction and intention to complete further courses with the JWL blended learning system, were articulated positively by students, a general fit of the setting to the requirements of the students can be assumed. In general, when comparing the results to those found by Zhang and Dang (2020), we could find significantly higher approval ratings for all variables in our study. While cultural biases might have impacted the results here (JWL reports on generally favorable outcomes of quantitative evaluations due to a culture of politeness and explicitly expressed appreciation in their target group), the overall results appear to indicate that the chosen implementation of blended learning meets student’s needs and requirements for HEI in marginalized regions.

As can be seen from the qualitative feedback, students valued the types of tasks and forms of learning such as interaction with class members in global discussion boards and flexible offline and multimedia learning packages significantly more than many other aspects of the JWL system. Consequently, the teaching method was addressed positively several times and explicitly referred to as particularly efficient or comfortable. These results clearly indicate that students in marginalized regions perceive the teaching method as crucial for the success of a learning system. The qualitative results thus back the assumption that the chosen blended learning approach can be considered suitable for the implementation of higher education measures for a target group of students in marginalized regions.

### Success factors from a technology-perspective

In numerous studies, the technically oriented D&M IS Success Quality dimensions, information quality, service quality and system quality have been examined with respect to the success of information systems, e-learning systems as well as in special studies on blended-learning systems. Not all of these studies found significant support for all three success factors (Mohammadi, 2015; Seman et al., 2019; Wu et al., 2010; Zhang & Dang, 2020). In the present study, we could show that of these factors, indeed only *information quality* had a significant impact on *task-technology-fit* and all other paths could not be confirmed to be relevant. When contrasting our results with those Zhang and Dang (2020), the role of *system quality* appears to have similarly impacted the dependent variables (in particular on *task-technology-fit* and *blended-learning flexibility*), whereas *service quality* was found to have significant impact on all three dependent variables by Zhang and Dang (2020). The lack of significant results and even a reverse effect on *learning climate* in our data, however, must not be over-interpreted, as the amount of data for service quality (n = 18) was too low to lead to meaningful results.

From a theoretical perspective, according to the findings of Ghazal et al. (2018a, b), the importance of *service quality* for success differs depending on the previous knowledge and training of the students with the system. As one student stated in his free response, and that is also described in the study by Ghazal et al. (2018a, b), tutorials or other retrievable support services are important from the students' point of view. Detailed training for students on how to use the system is considered even more beneficial. Intensive student training would avoid frustration while using the platform. If this is extensively fulfilled or if the students are already familiar with the system, it is possible that the service quality in an evaluation shows hardly any significant influence, in line with the results of Ghazal et al. (2018a, b).

Based on these findings, it can be concluded that *service quality* in the sense of support is important for the success of a blended-learning program from the perspective of students in marginalized areas. Intensive student training is therefore recommended (Ghazal et al., 2018a, b).

### Success factors from a pedagogical perspective

As mentioned at the beginning, not only aspects from the technical perspective play a critical role in blended-learning systems, but also factors that result from an educational or pedagogical perspective. *Computer Self-Efficacy* is a success factor of blended-learning systems, which is discussed very controversially in the literature. Some authors have found significant influence on aspects of e-learning system success in previous evaluations (Chen, 2014; Lee & Hwang, 2007; Wu et al., 2010), others like Al-Busaidi (2012) could not find that computer self-efficacy is a critical factor for success. This is in line with the results of the present study. A potential explanation of this phenomenon can be found in the background of the participants: students in developing countries have very different levels of computer literacy (Ghazal et al., 2018a, b) and therefore they might have very different levels of computer self-efficacy. This is also reflected in the fact that the *computer self-efficacy* factor shows the highest variance. Since the system is generally perceived as satisfactory, Ghazal et al. (2018a, b) conclusion that a well-designed system does not require specific technological skills could be seen as a reason why *computer self-efficacy* and intention to take further courses within this system do not correlate. This is also supported by the fact that the participants did not mention computer self-efficacy as an issue in the free answers or suggestions for improvement. The results of Zhang and Dang (2020) show a coherent picture here, as they too did not find any significant effects of *computer self-efficacy*.

While student’s *motivation* shows the highest overall rating of the independent variables in our dataset, we could not identify any significant effects on any of the dependent variables. This differs from the results of Zhang and Dang (2020), as they could find significant impacts on learning climate and task technology fit. Motivational aspects are also not mentioned in the qualitative part of the study, indicating that individual *motivation* might be considered a prerequisite for students in marginalized communities rather than a factor that impacts blended learning success.

*Media richness* of learning content as a pedagogical aspect does not expose any significant effects on the dependent variables. This again differs from the results of Zhang and Dang (2020), as they could find significant impacts on learning climate and task technology fit. One notable result, though, is a (non-significant) negative effect of *media richness* on *blended learning flexibility* we found in our data. When triangulating with the qualitative data, we can assume that due to the challenging infrastructural situation, multimedia content (such as videos) potentially has a negative impact on the perceived blended learning flexibility, as access to media-rich content might be bound to certain locations or points in time. While in particular the qualitative data hints at very positive effects on learning climate and task-technology fit, we could not confirm such effects in the quantitative data. Deliberately designing *media richness* in learning content thus seems to be a critical trade-off, that needs to be considered due to its conflicting effects on higher-level blended learning success factors for students in marginalized regions.

The only independent variable that shows significant positive impact on all three dependent variables is *teaching method*. This is consistent with the results of Zhang and Dang (2020), although the path coefficients are even higher in our data. This points at the importance of the teaching method for students in marginalized communities. This is also backed by the qualitative data, which only shows comments with a positive sentiment in this category.

### Success factors from an integrated blended learning perspective

Zhang and Dang's (2020) model includes three constructs that have been shown to be significant in the current research literature on success factors of e-learning or blended learning systems: *learning climate*, *task-technology fit* (Lin & Wang, 2012; McGill & Klobas, 2009) and *blended-learning-flexibility* (Al-Busaidi, 2012; Ghazal et al., 2018a, b).

In the evaluation conducted in this paper, *blended-learning flexibility* showed a significant impact on the *intention* factor, whereas *satisfaction* was not significantly impacted. *Learning climate* showed significant impacts on both*, satisfaction* and *intention*. *Task-technology fit* had a significant impact on *satisfaction* only. These results give an indication that students in marginalized regions consider these three factors as blended-learning success factors to different extents. Looking at the mean values for the variables in the quantitative part of the survey, *task-technology fit* has shown the lowest agreement for the examined system. Considering that this factor shows significant impact on satisfaction of a blended-learning system, it could be concluded that further development of the system is necessary to improve the task-technology fit.

Summarizing the results based on Zhang and Dang’s (2020) model, the variables *information quality*, *teaching method, learning climate, task-technology-fit* and *blended-learning flexibility have* shown significant impact on their respective dependent variables to different extents and thus in general should be considered relevant factors for blended-learning system success from the perspective of students in marginalized regions. In addition, from triangulation with the qualitative data, *service quality* and *media richness* are factors that are perceived considerably different than in traditional HEI settings as examined by Zhang and Dang (2020) and thus also should be deliberately considered when designing a blended-learning setting for students in marginalized regions.

### Additionally identified success factors

Facilitator-related aspects have a major impact on the success of a blended-learning system and should therefore also be seen as a success factor. Numerous authors have already identified significant influence of facilitator characteristics on the success of the blended-learning system in their research (Cheng, 2012; McGill & Klobas, 2009; Seman et al., 2019).

In the research model of Zhang and Dang (2020), this factor was not explicitly included.

Students’ responses in the open questions show that the aspect of online facilitator communication should be considered, as well as online facilitator response timelines and online facilitator feedback quality. Furthermore, the results show that the aspects onsite facilitator support and onsite facilitator qualification, moderation skills and helpfulness and guidance to use the system should also be considered as crucial impact factors of the onsite facilitator on student satisfaction. This clearly demonstrates that from the perspective of students in marginalized regions, the facilitator characteristics factor has an impact on the success of the blended-learning system. From these results it can be seen that, from the perspective of students in marginalized regions, the *facilitator characteristics* factor is critical for the success of blended-learning systems.

Numerous publications indicate that comprehensive facilitator training is essential to prepare facilitators to perform these critical tasks to the satisfaction of students (Alomari et al., 2020; Ghazal et al., 2018a, b). Thus, a substantial *facilitator training* can also have an impact on the success of a blended-learning system.

From the perspective of students in marginalized regions, the extent to which the necessary hardware and internet connections are available is also critical to the success of the blended learning system. The students' statements on this can be summarized in three aspects: *accessibility of hardware* for studying, *fit of available hardware* to the requirements in the learning system and *accessibility of internet connectivity* that fulfills the requirements of studying with the blended-learning system. By adapting the equipment of the learning centers to these aspects, facilitation can help students to be more satisfied with the system. However, these findings are still very vague and therefore not very reliable and should be further investigated in future studies.

In addition to the factors already mentioned in Section 5.3, the students surveyed also see the factors *facilitator characteristics* and *hardware and internet connectivity* as critical success factors for blended-learning systems. For each of these factors, various aspects were found that should be considered when enrolling further courses or planning further offers for students in marginalized regions worldwide to establish a successful blended-learning system.

## Conclusions

This article explored the question of the success criteria of a blended-learning system for students living in marginalized regions globally. The results of the study will help to facilitate access to higher education for students in marginalized regions by establishing criteria for future blended-learning system implementations that reflect the success factors from the perspective of the target group. In the widest sense, this can also contribute to UNHCR's goal of enrolling 15% of young refugees in higher education programs by 2030, as one strategy to achieve this goal is the expansion of connected learning programs. An established provider of these programs is JWL and therefore a case study was conducted at JWL to investigate what success factors should be considered in these programs.

What can be achieved with successful blended-learning systems, besides contributing to the UNHCR goal, is shown by the feedback written for the JWL courses by a student in the survey of this research:

*“The courses you offered gives one to know his/her identity. it led to have positive moral values in the community and outside therefore, you can be a good example for others especially youths who are addicted to drugs abuses. It help[s] to gain knowledge and transform the society as an extra-curricular education. Help to express depression and can reaching to the solutions easily. It can be good if you offer higher education to widening the skills and become perfect intellectual so that to be easy in tackling tough conditions by helping those who are in depression.”*

As this quote indicates and the survey data shows, the JWL system can be seen as an example of a successful blended-learning system, as it is highly approved by students and many of them wish to take further programs of this kind. Students of JWL in different marginalized regions around the world saw their satisfaction with the system and their intention to take further courses as being influenced by several factors. On a more general level, we could identify the impact factors on blended learning success that appear to be particularly relevant when designing offers for students in marginalized regions. Of those factors identified to be relevant for blended learning success in HEI in general (Zhang & Dang, 2020), the *teaching method*, *information quality, learning climate, task-technology-fit* and *blended-learning flexibility* appear to be of particular importance. *Media richness* and *service quality* appear to be highly contextualized impact factors, which need to be designed for the specific setting in which the blended learning offers are aimed to be deployed to not cause any adverse effects.

Further, *facilitation characteristics* are critical success factors for students in marginalized regions around the world. Intensive and holistic training of the facilitators is recommended to reach a positive impact through this success factor. In addition, the investigated context showed that from the students' perspective, the *equipment with suitable hardware and sufficient internet connectivity* is critical for the success of the blended learning offer.

This work also has some limitations that need to be considered when interpreting the results. This research examined a highly specialized blended-learning program which is adapted to the challenges of students in marginalized areas where the organization JWL has been working for many years and has already gathered experience. That might have affected the generalizability of the results to a wider group of students in marginalized areas. Furthermore, the impact of other factors caused by the environment or JWL as organization were not taken into account but might have impacted the results. In addition, it should be noted that the students, as indicated by the demographic survey, mostly came from countries in which people often refrain from openly criticizing issues for cultural reasons. This in turn could have led to a bias in the data, which we have accounted for by taking the overall average score as a baseline for interpretation.

To improve the validity of the data, the *service quality* in particular should be re-evaluated in further research steps, as the small sample is not a reliable basis for interpretation. The same applies to the *facilitator characteristics* and *hardware and internet connectivity aspects*, as these were examined only in the qualitative part of the study. In addition, the study should also be conducted in other contexts from the perspective of students in marginalized regions to exclude possible biases introduced by the structure of JWL’s offers and to thus obtain more generalizable data. If these studies were conducted within a larger population, it might be possible to draw regional or cultural conclusions.

With these findings, future blended-learning systems could be better adapted to the target groups. With more and more adapted programs, satisfaction, and intention to take courses would rise and more equality in higher education can be created in the global context. In the widest sense, this can then also contribute to UNHCR's goal of enrollment of 15% of refugee youth in higher education programs by 2030.

## Data Availability

The datasets used and analyzed during the current study are available from the corresponding author on reasonable request.

## Annex 1 – Items of questionnaire

**Table:**

Computer Self-Efficacy		
I enjoy using computers and tablets		CSE1
I am confident about using computers and tablets		CSE2
In general, I am comfortable with using computers, tablets and software applications		CSE3
Motivation		
The blended learning environment motivates me to learn the course content	M1	
I feel my motivation to learn the course content increases because it’s offered as a blended-learning program. (Compared to a course in complete online self-directed learning or complete In-Class mode)	M2	
The In-Class Meetings enhance my motivation in studying this course	M3	
Information Quality		
The information provided through the Learning Platform is relevant for my learning. (Help: I can see that the learning materials are relevant to reach the learning objectives of this course. The information on the platform is relevant to do the learning activities and assignments successfully.)	IQ1	
The information provided through the Learning Platform is easy to understand. (Help: The language used is easy to understand. The learning materials are presented in a way that I can easily understand the information)	IQ2	
The information provided through the Learning Platform is overall very good	IQ3	
The information provided through the Learning Platform is up to date. (Help: The examples, case studies and materials seem to be up to date (Not outdated and old))	IQ4	
The information provided through the Learning Platform is complete. (Help: There are now open questions, after studying one unit I know how to do the activities and assignment.)	IQ5	
The information provided through the Learning Platform is accurate. (Help: The information is correct in all details)	IQ6	
System Quality		
The technology of the Learning Platform offers flexibility in learning as to time and place	SQ1	
The technology of the Learning Platform offers multimedia (audio, video, and text) types of learning materials	SQ2	
The technology of the Learning Platform offers sufficient functions for my learning	SQ3	
In general, the technology of JWL’s Learning Platform is reliable	SQ4	
In general, response time of the technology of the Learning Platform is reasonable	SQ5	
Service Quality		
Have you ever contacted the JWL-Helpdesk (Mail address: jwl-helpdesk@seitwerk.de)?	SVQ0	
The Helpdesk for JWL’s Learning Platform answers promptly. (Help: Answers within 72 h)	SVQ1	
The Helpdesk for JWL’s Learning Platform is reliable	SVQ2	
The Helpdesk for JWL’s Learning Platform is accessible. (Help: is accessible (= can be contacted easily))	SVQ3	
Using the Helpdesk to get support is convenient	SVQ4	
Overall, my impression of contacting the Helpdesk for JWL’s Learning Platform is satisfactory	SVQ5	
Media Richness		
In addition to the In-Class Meetings (via Zoom or Onsite), the blended learning course also uses a wide range of online media to support my learning, including text, images, audio, animations and videos	MR1	
The various types of online media (including text, images, audios, animations and videos) used in the blended learning course are effective for my learning needs	MR2	
The various types of online media (including text, images, audio, animations and videos) used in the blended learning course are helpful for me to complete the assignments and learning activities	MR3	
Overall, the blended learning course provides a wide range of media that are supportive for my learning	MR4	
Teaching Method		
I have a positive feeling toward using the digital learning materials during the In-Class Meetings	TM1	
The interactive self-study materials are helpful for me to learn the content input of each unit	TM2	
Having several small learning activities is helpful for me to learn the concepts covered in the unit	TM3	
Assignments and Project work are helpful to find out if I´m able to transfer what I have learned to real tasks	TM4	
The exchange with my classmates in the global class discussions is helpful to deepen my understanding of the unit’s learning content	TM5	
The In-Class Meetings each week are useful to help me learn the course subjects	TM6	
Having practical relevant assignments and activities where I bring in the experiences of my community keeps me motivated to keep studying this course	TM7	
Learning Climate		
The process of using the JWL Learning Platform to assist my learning is pleasant	LC1	
I have fun with the blended learning program	LC2	
I find the blended learning program to be enjoyable	LC3	
The learning climate provided by the blended learning program could motivate my spontaneous learning	LC4	
Task-Technology-Fit		
The various functionalities used on the Learning Platform for submitting the activities and assignments match my learning needs	TTF1	
The technology of the Learning Platform suits for all tasks I have to submit while studying this course	TTF2	
This course provides me with all the information and material I need to reach my learning goals	TTF3	
I feel that my learning goals and needs are met by utilizing JWL’s Learning Platform for blended learning programs	TTF4	
Blended- Learning Flexibility		
Meeting my onsite facilitator only twice a week in the classroom and working on the activities and assignments at my own place of choice (either in the learning center or at home) gives me more flexibility to study this course	BLF1	
Meeting my onsite facilitator only twice a week in the classroom and working on the activities and assignments at my own place of choice (either in the learning center or at home) allows me to learn and complete the assignments at my own pace	BLF2	
Meeting my onsite facilitator only twice a week in the classroom and working on the activities and assignments at my own place of choice (either in the learning center or at home) allows me to arrange my study time for the course more effectively	BLF3	
Satisfaction		
Overall, the blended learning course is enjoyable	SAT1	
Overall, the blended learning program is pleasant to me	SAT2	
This blended learning course gives me self-confidence	SAT3	
Overall, the blended learning environment satisfies my educational needs	SAT4	
Intention		
If available, I intend to take more blended learning courses in the future	INT1	
I´m likely to attend another blended-learning course in the near future	INT2	
Other		
Do you have any further ideas how JWL Courses and Learning Platform can be improved? What kind of courses would you like to be offered to satisfy your learning needs? What needs to be improved on the learning platform? Your ideas and comments are highly appreciated		

## Annex 2 – Deductive-inductive coding of qualitative results

The numbers in brackets in the right column refer to the ID of survey participants. Of the 80 students who filled in the survey, 19 did not respond to this question and the remaining 61 wrote a comment in which they addressed success factors or suggested improvements to the system. The addressed aspects were thematically coded using a deductive approach (Braun & Clarke, 2006) following the categories used in the research model of Zhang and Dang (2020). Since many statements also contain several aspects, they are also assigned to several categories. For those comments, which could not be coded using the existing scheme of categories, inductive coding was applied in a second step (Braun & Clarke, 2006). The additional categories are listed in a separate category in the table below.

**Table:**

Deductive coding results	
Information Quality (7x)	(21) Programm is relevant for my learning (35) Ami kindly appreciate the important of JWL to engage us in skills and experience my opinion so since well and enjoying alat (54) It helps me a lot to improve my self-confidence (57) if we know about the facilitators expectation only we can give our correct answer so if you include it will be good we have enough materials to do our assignments easily (106) The courses you offered gives one to know his/her identity. it led to have positive moral values in the community and outside therefore, you can be a good example for others especially youths who are addicted to drugs abuses. It help to gain knowledge and transform the society as an extra -curricular education. Help to express depression and can reaching to the solutions easily. It can be good if you offer higher education to widening the skills and become perfect intellectual so that to be easy in tackling tough conditions by helping those who are in depression (141) First of all, I want to thank the JWL committee for providing us with such useful courses that help us to improve and expand our knowledge and understanding All the courses are useful and needed for us to learn them (153) By attending JWL Course and Learning Platform is very benefit, very helpful and improving by discovering about myself
Media Richness: (6x)	(18) Videos are more fun and understandable (28) Fully satisfied with my learning needs, sometimes reading is difficult (48) More videos would be great for my understanding (52) the books and Pdf its such old and boring style, (the videos of) Dr barbara make me get the idea in a few minutes that what attracts us more (81) Use more videos than PDFs, Reading cases is boring (127) Since its blending learning, there should be some more video and audio part in the portal,it would be better and more helpful
System Quality: (3x)	(29) JWL learning platform is more useful for the students, who are living in the poor (127) The portal should be easy to understand, the learner could find every unit and detail easily (70) The course and the platform is well designed only to say that it becomes hard when new students are starting the course because it the thing which we were not familiar with but as we keep on studying we get used and all goes smoothly
Service Quality: (1x)	(127) This learning program should have a guide or a demo how to use the material and portal
Teaching Method: (4x)	(59) No time limit for Assignments to submit (85) Blended-learning approach makes me feel comfortable, confident and meets my learning needs (103) the program has a lot of impact on me. Its very effective and useful. I learned and also improved the way of teaching (151) I like JWL courses and learning Porcess that is very effective and helps me to improve
Categories identified via inductive coding	
Hardware: (6x)	(37) You may gave us big lap top which are new (38) Firstly, in order to improve JWL's learning platform we need to be given tablets or computers so that for is refugees it should be easier because we spend much time while trying to borrow smart phones or computers to use, so if we are provided with laptops or tables it can make our work easier (51) There should be laptops or tablets available for these blended learning programs (71) Treat everyone as equal. I mean when it comes to devices(computer, tablet and so on) (115) I think it would be better if your center provide a better internet connection (140) If we could have the har of the reading or any tablet to watch the videos offline it will be great
Facilitator Characteristics general: (2x)	(51) There should be active communication between Students and online facilitators because once we get stuck or have questions, we write questions to the online facilitators but no replies. It could be good to have feedback on each work submitted within a unit or week. Yet, weekly works which are to be marked by the online facilitators are delaying with marking them. We only get feedback from works which are under responsibility of the Onsite facilitator (91) I can see my facilitator and classmates but one thing I would like to suggest that it would be better if we could have discussion face to face in the platforms with the specific time and schedule. Right now, we can communicate only by using text to reply and comment. We don't have the chance to see face to face and having verbal communication
Facilitator Characteristics onsite: (7x)	(25) I am very happy to participate in this course. specially I am thankful from Ms. Shallyn. She is a very kind and understandable girl (47) there is a suggestion that our onsight facilitator is very weak and doesn't have enough knowledge about the subject (53) overall the course contents are good but the course facilitator didn't guide properly and for the first two weeks we were not aware of how to access the course contents therefore it created confusion and it caused the delay in submission of assignment and project work (70) The course and the platform is well designed only to say that it becomes hard when new students are starting the course because it the thing which we were not familiar with but as we keep on studying we get used and all goes smoothly (72) Moreover, the InClass meetings don't fulfil my learning expectations because everything discussed are quite basic in nature and nothing new is learned so far (107) Advertisement is the best thinks for improve our course because here nobody not much know about this course (127) The facilitator should at least give a brief detail about how to use the material and portal. The facilitator should be helpful toward learners
Facilitator Characteristics online: (5x)	(29) what needs to be improved is the way of scoring the discussions and the feedback from the facilitator for each one (50) I would strongly suggest that there should be a team to check the knowledge of all the online facilitators before giving them a class to handle, as choosing weak online facilitator from the in charge centers can bring the quality of the lessons very low, and giving students bad learning experiences which will end up in not participating in the future learning courses (64) I am really, grateful for this wonderful, and unforgettable casualty. My online facilitator Ms Tsoi should be appreciated and regarded, for the support she rendered (73) what I wanted to ask if possible when marking our works if a person fails he or she needs your comment so that he may know what was the casuse of the failure (139) There should be an active collaboration between students and online facilitators because they don't give feedback on weekly submitted works. students cannot be able to know if they are doing the works correctly
